# Increase *Trichomonas vaginalis* detection based on urine routine analysis through a machine learning approach

**DOI:** 10.1038/s41598-019-47361-8

**Published:** 2019-08-19

**Authors:** Hsin-Yao Wang, Chung-Chih Hung, Chun-Hsien Chen, Tzong-Yi Lee, Kai-Yao Huang, Hsiao-Chen Ning, Nan-Chang Lai, Ming-Hsiu Tsai, Li-Chuan Lu, Yi-Ju Tseng, Jang-Jih Lu

**Affiliations:** 1 Department of Laboratory Medicine, Chang Gung Memorial Hospital at Linkou, Taoyuan, Taiwan; 2 Ph.D. Program in Biomedical Engineering, Chang Gung University, Taoyuan, Taiwan; 3 School of Medicine, Chang Gung University, Taoyuan, Taiwan; 4 Department of Medical Biotechnology and Laboratory Science, Chang Gung University, Taoyuan, Taiwan; 5 Graduate Institute of Technological and Vocational Education, National Taipei University of Technology, Taipei, Taiwan; 6 Department of Laboratory Medicine, Taipei Hospital, Ministry of Health and Welfare, New Taipei City, Taiwan; 7 Department of Information Management, Chang Gung University, Taoyuan, Taiwan; 8 Department of Computer Science & Engineering, Yuan Ze University, Taoyuan, Taiwan; 9 Innovation Center for Big Data and Digital Convergence, Yuan Ze University, Taoyuan, Taiwan; 10 Warshel Institute for Computational Biology, Chinese University of Hong Kong, Shenzhen, China; 11 School of Science and Engineering, Chinese University of Hong Kong, Shenzhen, China; 12 Department of Pathology, National Defense Medical Center, Division of Clinical Pathology, Tri-Service General Hospital, Taipei, Taiwan; 13 Research Center for Emerging Viral Infections, Chang Gung University, Taoyuan, Taiwan

**Keywords:** Parasitic infection, Translational research, Risk factors, Diagnostic markers

## Abstract

*Trichomonas vaginalis* (*T*. *vaginalis*) detection remains an unsolved problem in using of automated instruments for urinalysis. The study proposes a machine learning (ML)-based strategy to increase the detection rate of *T*. *vaginalis* in urine. On the basis of urinalysis data from a teaching hospital during 2009–2013, individuals underwent at least one urinalysis test were included. Logistic regression, support vector machine, and random forest, were used to select specimens with a high risk of *T*. *vaginalis* infection for confirmation through microscopic examinations. A total of 410,952 and 428,203 specimens from men and women were tested, of which 91 (0.02%) and 517 (0.12%) *T*. *vaginalis*-positive specimens were reported, respectively. The prediction models of *T*. *vaginalis* infection attained an area under the receiver operating characteristic curve of more than 0.87 for women and 0.83 for men. The Lift values of the top 5% risky specimens were above eight. While the most risky vigintile was picked out by the models and confirmed by microscopic examination, the incremental cost-effectiveness ratios for *T*. *vaginalis* detection in men and women were USD$170.1 and USD$29.7, respectively. On the basis of urinalysis, the proposed strategy can significantly increase the detection rate of *T*. *vaginalis* in a cost-effective manner.

## Introduction

Trichomoniasis, which is caused by *Trichomonas vaginalis* (*T*. *vaginalis*), affects 30.1 million people in the World Health Organization (WHO) Western Pacific Region and 187.0 million people globally, making it the most prevalent nonviral sexually transmitted infection^1^. *T*. *vaginalis* has been reported to cause several human infections, typically of the urogenital organs, such as vaginitis, urethritis, and prostatitis^2^. The symptoms of *T*. *vaginalis* infection vary; up to 50% female patients exhibit no symptoms^2^. *T*. *vaginalis* infection causes some classical urinary tract infection-like symptoms, such as dysuria and urinary frequency and urgency^3^. Two-thirds of infected individuals remain undiagnosed and untreated^4^. Untreated individuals sustain potential infections lasting from months to years^4^. Typically, this infection does not result in serious sequela. However, in recent years, a number of studies reported some virulence factors of *T*. *vaginalis* that are associated with severe consequences^5–7^. It means that *T*. *vaginalis* is not always a self-limited infectious disease. Trichomoniasis has been reported to be related to prostate^8–12^ and cervical cancer^13–15^, premature birth^16,17^, and infertility^18^. Furthermore, undiagnosed/untreated trichomoniasis is associated with crucial public health concerns. Despite the importance of *T*. *vaginalis* infection, accurate diagnosis of *T*. *vaginalis* infection is yet to be standardized.

Trichomoniasis diagnosis can be enhanced by an adequate screening tool^1,17,19^. However, the cost-effectiveness of screening asymptomatic individuals for *T*. *vaginalis* infection has not been sufficiently addressed^1,17^. *T*. *vaginalis* can be detected using various test methods, including the microscopic examinations of urine sediments, wet preparation of genital secretions, polymerase chain reaction (PCR), and antigen–antibody rapid screening. Wet preparation of genital secretions or wet mount is the diagnostic method of choice recommended by the Centers for Disease Control and Prevention. The wet mount method includes specimen collection from the vagina or urethra followed by staining and microscopic examination^4^. A good wet mount test largely depends on the adequate collection of vaginal discharge, which should be performed by well-trained medical staff. Consequently, the compliance of wet mount is restricted by limited medical staff. PCR methods^20,21^ and antigen–antibody rapid screening^22^ could detect *T*. *vaginalis* with high accuracy. However, the availability and cost-effectiveness of these tests limit their use in routine diagnostic laboratories^23^. By contrast, the microscopic examination of urine remains one of the most commonly used methods for *T*. *vaginalis* screening^24^, although it is less sensitive than other methods^25^. Microscopic examination of urine is a part of routine urinalysis test, which also tests a number of items, including leukocyte esterase, nitrite, protein, occult blood, red blood cell (RBC) count, white blood cell (WBC) count, epithelial cell count, and sediments in urine specimens. To date, universal microscopic examinations for urine sediments seem to be impractical because of the increasing specimens and limited medical resources^18^. A visual examination of every specimen by medical technologists is an extremely labor-intensive and time-consuming task^19^. Therefore, to examine overwhelming specimens, the routine urinalysis test was automated. Automated instruments can shorten the processing time, reduce the requirement of human resources, and considerably improve efficiency^20–23^. However, all these automated instruments have limitations in detecting *T*. *vaginalis*^23,24^. Prior to the use of automated instruments, the detection rate of *T*. *vaginalis* achieved through the visual examination of every urine sediment specimen was approximately 0.1%, based on the data obtained from Chang Gung Memorial Hospital (CGMH), Linkou branch. The detection rate dropped to almost zero, and the role of *T*. *vaginalis* screening was compromised after the introduction of automated instruments for urine sediment examinations.

Thus, given the fact that all of the routine urinalysis tests have been automated, we intended to improve *T*. *vaginalis* detection on the basis of other urinalysis test results (i.e., leukocyte esterase, nitrite, protein, occult blood, and RBC, WBC, and epithelial cell counts). We hypothesized that a specific pattern of urinalysis test results for *T*. *vaginalis*-positive cases versus *T*. *vaginalis*-negative cases would be noted. To address the pattern recognition problem, we utilized machine learning (ML) algorithms trying to identify the specific pattern of urinalysis test results in *T*. *vaginalis* infection. ML methods are algorithms that can classify unknown cases by learning the multivariable pattern of training cases^26^. The successful application of ML algorithms in biomedical research or clinical use has been reported and raised considerable attention in recent years. Most of the applications were reported in the field of radiology^27^, dermatology^28^, ophthalmology^29^, oncology^30,31^, and anatomic pathology^32^. Still others utilized ML algorithms in analyzing mass data in the field of microbiology^33–35^. To date, however, only a few applications of ML algorithms for analyzing real-world laboratory data have been published^26,36^. The study would be the first to use the routine urinalysis data to increase *T*. *vaginalis* detection. In this study, we developed a strategy for optimizing specimen selection for microscopic examinations to facilitate *T*. *vaginalis* detection. We propose an ML-based strategy for predicting *T*. *vaginalis*-infected specimens based on the data obtained from automated urinalysis. Through the use of our ML-based strategy, the detection rate of *T*. *vaginalis* could be increased in a cost-effective manner.

## Results

### Patient characteristics and urinalysis results

A total of 410,952 and 428,203 specimens from men and women were tested, of which 91 (0.02%) and 517 (0.12%) *T*. *vaginalis*-positive specimens were reported, respectively. Table 1 presents the demographic characteristics and urinalysis results. *T*. *vaginalis*-positive specimens were more likely to have a higher level of leukocyte esterase and protein, as well as higher WBC and epithelial cell counts, in both women and men, compared with *T*. *vaginalis*-negative specimens (p < 0.001). Women with *T*. *vaginalis*-positive specimens were younger than those with *T*. *vaginalis*-negative specimens. By contrast, men with *T*. *vaginalis*-positive specimens were older than those with *T*. *vaginalis*-negative specimens. The distributions of continuous and noncontinuous urinalysis test results were showed in Supplementary Figs 1, 2, respectively.

**Table 1:** Demographic Characteristics and Urinalysis Results of Individuals with *Trichomonas vaginalis*-Positive and *Trichomonas vaginalis*-Negative Specimens.

	Women			Men		
	Trichomonas (+)	Trichomonas (−)	P value	Trichomonas (+)	Trichomonas (−)	P value
Patients, n	517	427,686		91	410,861	
Age, mean (SD^a^)	43.0 (15.7)	47.9 (23.0)	<0.001^c^	63.5 (13.5)	48.5 (24.3)	<0.001^c^
Leukocyte esterase, median (IQR^b^)	3 (2)	0 (0)	<0.001^d^	1 (2)	0 (0)	<0.001^d^
Nitrite, n (%)	35 (6.8)	35987 (8.4)	0.20^e^	7 (7.7)	19,131 (4.6)	0.26^e^
Protein, median (IQR)	0 (2)	0 (1)	<0.001^d^	1 (3)	0 (2)	<0.001^d^
Occult blood, median (IQR)	1 (3)	0 (2)	<0.001^d^	0 (2)	0 (2)	0.09^d^
RBC, mean (SD)	54.4 (128.4)	34.4 (103.0)	<0.001^c^	37.6 (114.6)	37.7 (112.2)	1.00^c^
WBC, mean (SD)	129.1 (159.4)	47.2 (113.8)	<0.001^c^	81.6 (141.4)	26.2 (90.1)	<0.001^c^
Epithelial cell, mean (SD)	33.1 (30.5)	9.6 (16.8)	<0.001^c^	8.5 (15.9)	1.6 (5.2)	<0.001^c^

^a^SD: Standard deviation; ^b^IQR: Interquartile range; ^c^Student t test; ^d^Mann–Whitney U test; ^e^Pearson chi-squared test.

### Model performance

**Figure 1:**
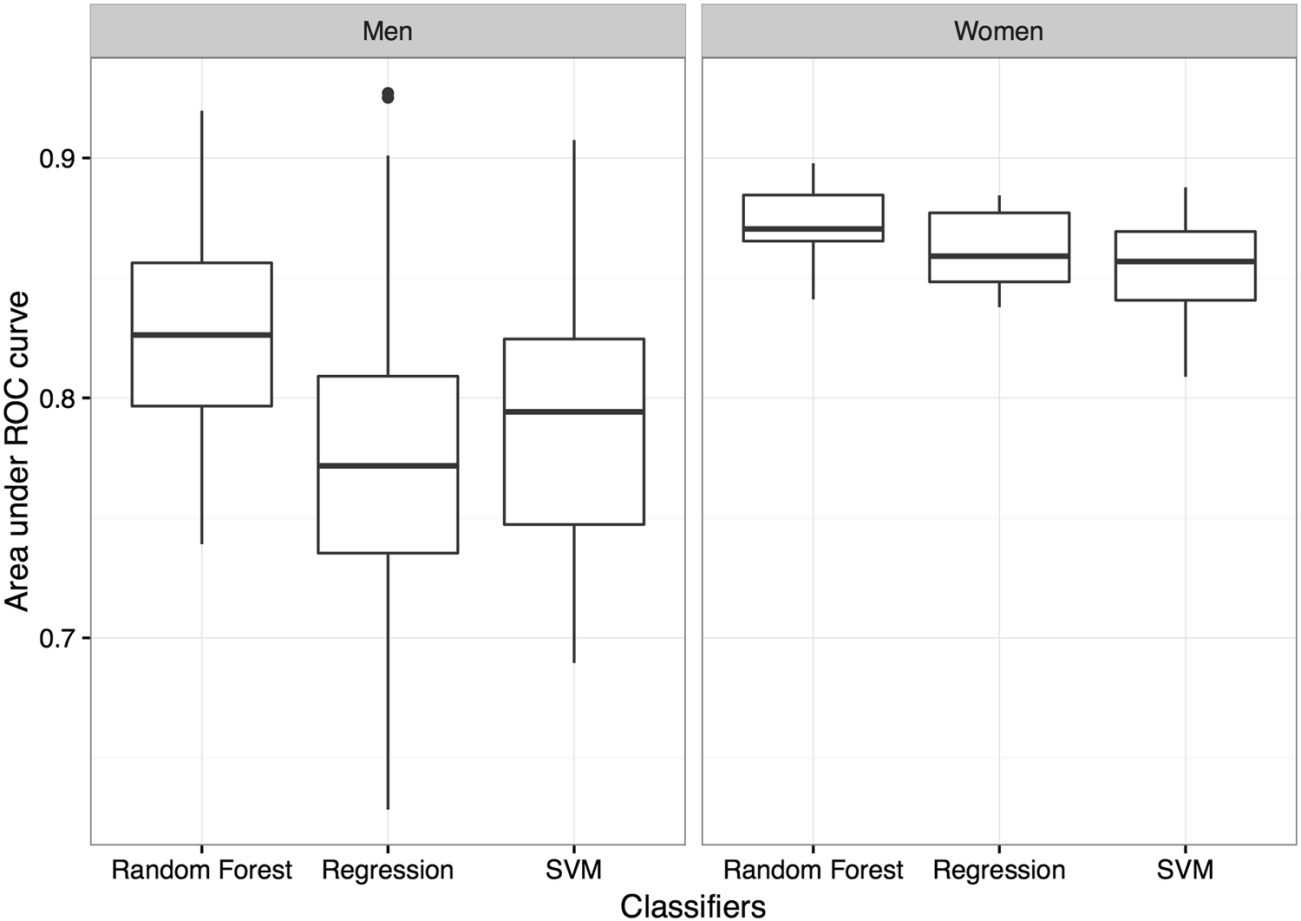
Performance of classification models constructed using random forest, linear regression, and SVM classifiers. ROC: Receiver operating characteristic, SVM: Support vector machine.

**Figure 2:**
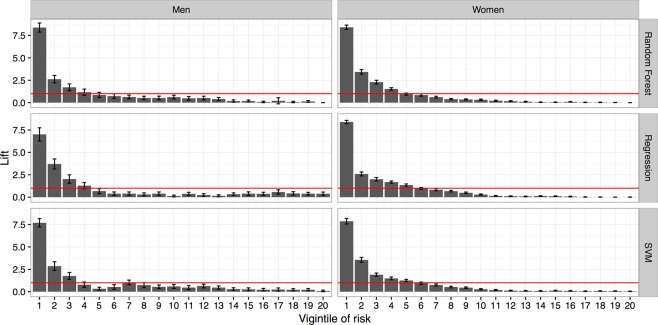
Vigintile-wise Lift chart of classification models for men and women. The horizontal lines in the subfigures indicate the average positive predictive value. SVM: Support vector machine.

For *T*. *vaginalis* detection, the most favorable models for the testing set (50 times, 5-fold cross validation with 10 randomly selected training dataset, Supplementary Fig. 3) were constructed using random forest, with the corresponding area under the receiver operating characteristic (ROC) curve (AUC) values being 0.87 and 0.83 for women and men, respectively (p < 0.001, Fig. 1). The result shows that the random forest model is a very good model for *T*. *vaginalis* detection^37,38^. The AUC values derived for multivariable models constructed using random forest, linear regression, and support vector machine (SVM) were significantly higher (p < 0.001) than those derived for single-variable models (Supplementary Table 1). Figure 2 shows prediction models’ Lift values in each vigintile. The Lift values in the highest vigintile of risk were 8.41 and 8.38 for women and men, respectively, indicating that the positive predictive value in the highest vigintile of risk was more than eight times higher than the average positive predictive value.

### Variable importance

**Figure 3:**
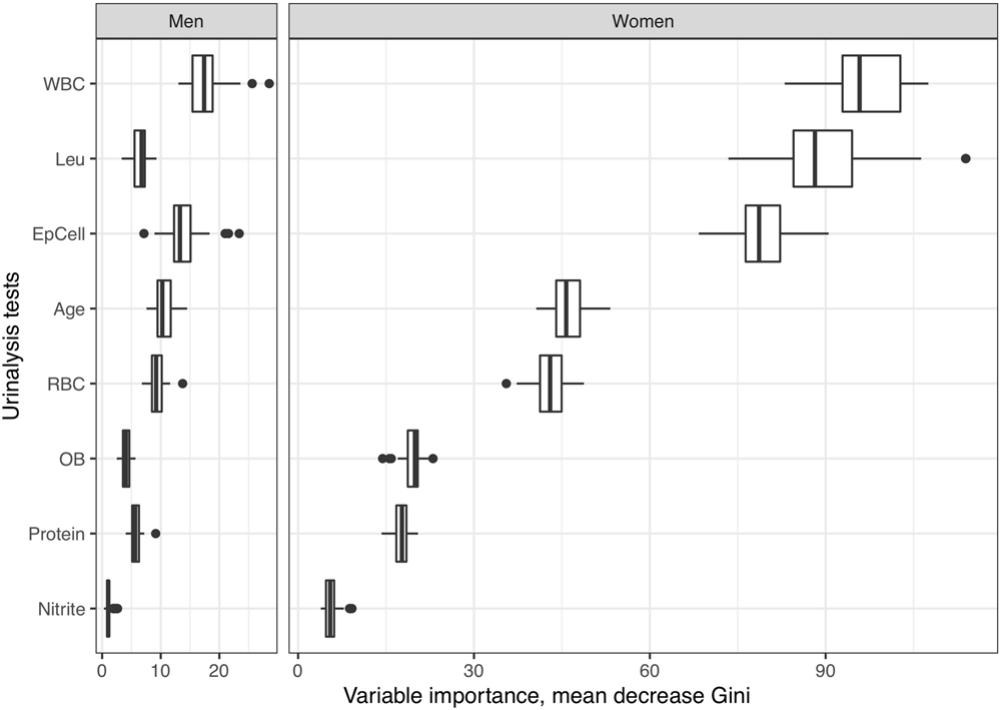
Variable importance of the optimal random forest model, defined by the mean decrease in Gini.

In the optimal random forest models for *T*. *vaginalis* detection in women, the most important urinalysis tests are leukocyte esterase, and WBC and epithelial cell counts (Fig. 3). The average mean decrease in Gini of these features was much higher than the other features in urinalysis tests. The features used in the model for men are similar (Fig. 3). The most important features were age, and WBC and epithelial cell counts. Leukocyte esterase was less important in the model for men. Although the RBC count is not significantly different between *T*. *vaginalis*-positive and *T*. *vaginalis*-negative specimens, the importance of the RBC count in the models were similar with age, contributing to the machine learning models.

### Cost-effectiveness analysis

**Figure 4:**
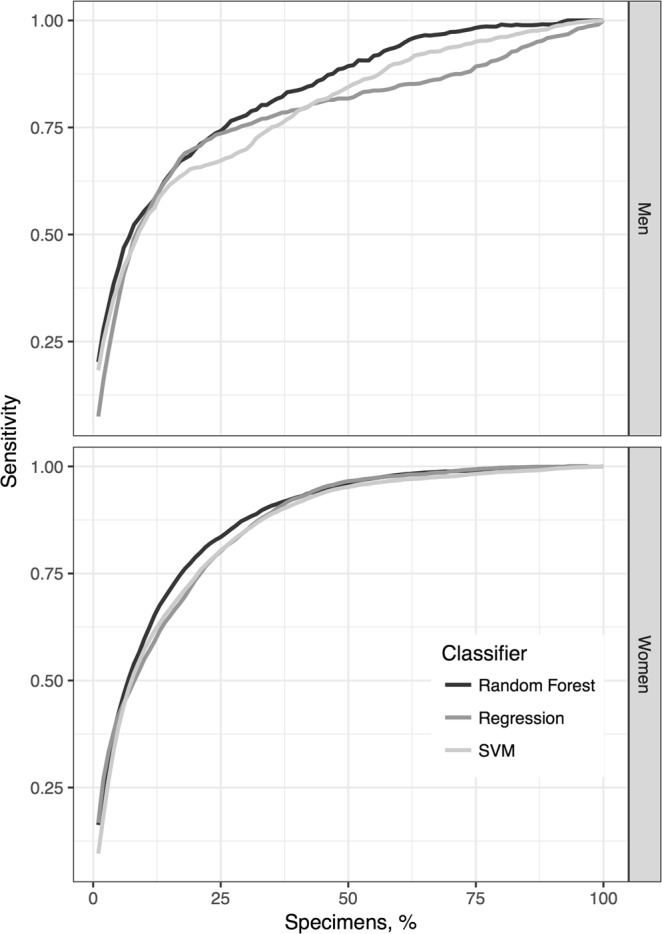
Curves showing sensitivity achieved by classification models at different percentages of microscopically examined specimens.

**Figure 5:**
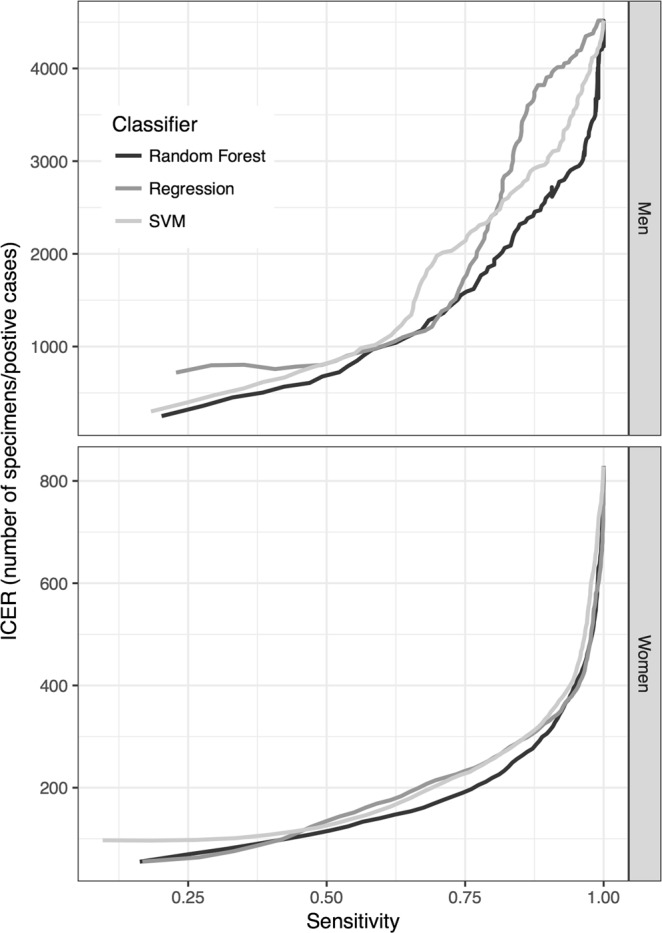
Curves showing ICERs at different sensitivity levels. The ICER is defined as the number of specimens microscopically examined divided by the number of positive cases found. ICER: incremental cost-effectiveness ratio.

Figure 4 shows plots of the sensitivity versus the percentage of specimens confirmed by microscopic examinations. The sensitivity levels of the classification models constructed using random forest were 83.5% and 74.2% in microscopically examined specimens of women and men, respectively, in the highest quartile of risk. Figure 5 shows plots of the incremental cost-effectiveness ratio (ICERs), the number of specimens microscopically examined divided by the number of positive cases found, versus the sensitivity. The ICERs demonstrated the cost (number of specimens tested) at different levels of effectiveness (number of positive cases found). At a sensitivity threshold of 75%, the lowest ICERs were 197.4 and 1591.4 for women and men, respectively, for the models constructed using random forest.

## Discussion

Urine specimens from individuals at a relatively high risk of *T*. *vaginalis* infection were successfully identified by the proposed detection model by using data from routine clinical practice. The performance and ICER evaluations revealed that our ML-based strategy can significantly increase the detection rate of *T*. *vaginalis* in a cost-effective manner.

Automation of urine sediment examinations can increase throughput and decrease labor. However, automated instruments could fail to detect some objects including *T*. *vaginalis*^39,40^. *T*. *vaginalis* detection methods have received considerable attention because this parasite is associated with various diseases^1,41^. The study variables obtained from automated urinalysis can be used for facilitating *T*. *vaginalis* detection (Table 1). However, the detection performance associated with using a single variable was not adequately satisfactory (Supplementary Table 1). By contrast, multivariable analysis may result in improved performance, because multivariable analysis provides more information than univariable analysis^26^. The ML models can serve as a sophisticated decision support tool for detecting *T*. *vaginalis* in urine specimens through training and validation by using a considerably large amount of data.

The data used in this study were collected from routine clinical practice. We used all features of urinalysis test to develop and evaluate the ML-based *T*. *vaginalis* detection strategy and did not exclude the tests which were not significantly different between *T*. *vaginalis*-positive and *T*. *vaginalis*-negative specimens because these variables could be important in the full multivariable model^42^. The label of every case was confirmed through microscopic examinations performed by sophisticated medical technologists with annual capacity assessment and College of American Pathologists (CAP) Laboratory Accreditation Program. Although the quality of data was optimized as much as possible, the possibility of false negative cases could not be completely excluded. A confirmatory test for *T*. *vaginalis* was not performed for several reasons. First, performing nucleic acid-based confirmatory tests for all specimens (i.e., approximately 800,000 cases in 5 years) would have been costly. Moreover, the ML algorithms used in this study are noise-resistant. Therefore, these methods can tolerate falsely labeled cases to some extent^43–45^. Consequently, the data were considered to be qualified for training robust ML models. All ML models achieved an AUC value of more than 0.85 in women (Fig. 1). The performance is sufficient to allow model application in clinical practice. By contrast, the performance of the ML models in men was lower than that in women. The lower AUC and higher standard deviation may have resulted from much fewer positive cases in men (91 cases) than those in women (517 cases; Table 1). As we known, a robust machine learning model depends largely on data with high fidelity and a sufficient sample size. In this work, although the data were collected over a period of 5 years from a reference hospital, we collected only 91 *T*. *vaginalis*-positive male cases. The paucity of *T*. *vaginalis*-positive cases in the male population could be explained by the fact that trichomoniasis is a female-predominant infectious disease. More data are required to construct highly robust models for men.

Urine sediment tests performed in our hospital were not intended for detecting *T*. *vaginalis*. However, we intended to increase *T*. *vaginalis* detection by urine sediment screening because it is one of the most frequently requested tests in our hospital (160,000 test requests per year on average). The urine sediment test using microscopic examinations remains a practical method for *T*. *vaginalis* screening in clinical practice. The advantageous features of this test include easy specimen preparation, immediate results, and low cost^46^. Among the methods capable of detecting *T*. *vaginalis*, the urine sediment test is ordered more frequently^41^. Therefore, it may be an acceptable tool for *T*. *vaginalis* screening^25,46^. However, it is extremely labor-intensive. After automation of the urine sediment test in 2015 in CGMH, microscopic examinations have no longer been performed for *T*. *vaginalis* detection; hence, the *T*. *vaginalis* detection rate in urine specimens dropped to zero. In the proposed strategy, ML methods were used to score high-risk specimens. We selected only high-risk specimens, identified by ML models, for further microscopic examination. Because overwhelming specimens are received for the urine sediment test (e.g., more than 600 specimens per day in CGMH), a trade-off should be made between the detection rate and the specimens selected for confirmation. If the optimized thresholds of the models obtained from ROC curves are used, approximately 30% of all specimens should be microscopically confirmed. The current manpower in the Department of Laboratory Medicine of CGMH could provide microscopic confirmation for 5% of all specimens following model prediction. The vigintile-wise Lift chart (Fig. 2) revealed that the Lift values were higher than 8 at the first vigintile in both men and women. The classification models provided a much higher probability of detecting *T*. *vaginalis* than universal microscopic examinations. A review of the top 5% risky specimens demonstrated that a sensitivity of approximately 40% was achieved (Fig. 4; Supplementary Table 2). The results indicate that approximately 40% of the infected cases could be detected through microscopic examinations of one-twentieth of all specimens, thus reducing 95% of the workload. Moreover, under this circumstance of revising top 5% risky specimens, the ICERs were 567 and 99 for men and women, respectively (Fig. 5; Supplementary Table 2). In this study, the ICER was presented as the number of specimens over positive cases to facilitate its utility in different areas and situations. In CGMH, microscopic examinations of urine sediments cost approximately US$0.3 per test. Consequently, the costs were determined to be US$170.1 (i.e., 567 × 0.3) and US$29.7 (i.e., 99 × 0.3) per positive case in men and women, respectively. The gross domestic product (GDP) per capita of Taiwan was approximately US$23,000 in 2016^47^. Therefore, *T*. *vaginalis* detection using the proposed strategy could be considered to be very cost-effective, because the corresponding cost is much lower than the annual GDP per capita, according to the WHO guidelines^48^.

The present study has several limitations. *T*. *vaginalis* detection was conducted through the microscopic examination of urine sediments. *T*. *vaginalis* detection in vaginal fluids by using a nucleic acid-based test, such as that reported in the National Health and Nutrition Examination Survey^49^, could have yielded different results. Furthermore, the data in this study were obtained from patients in a reference hospital; the prevalence and incidence might vary with hospitals and regions. The prevalence observed in microscopically examined urine sediments in this study was approximately 0.1%, which is lower than that reported by the WHO or US Centers for Disease Control and Prevention^50^; this discrepancy could be attributed to the aforementioned reasons. The present study successfully demonstrated an ML-based approach for selecting high-risk specimens for further manual detection of *T*. *vaginalis*. To obtain a highly robust and reliable ML model applicable in clinical practice, nucleic acid-based confirmatory tests may be necessary in the future for assigning a highly accurate label to each urine specimen.

## Methods

### Study population and data

We performed a retrospective study using laboratory data collected from a 3,383-bed teaching hospital in Taiwan (Chang Gung Memorial Hospital, CGMH) between January 2009 and December 2013. Individuals who underwent at least one urinalysis test [including urine chemistry (URISYS 2400, Roche Diagnostics Corp., Indianapolis, IN, USA) and urine sediment tests through microscopic examination] were included. Cases of *T*. *vaginalis* infections were identified according to positive *T*. *vaginalis* test results obtained from microscopic examinations. Individuals with negative test results were considered as controls. If individuals had multiple test results in the study period, all results were included in the analysis. The urinalysis test results consisted of *T*. *vaginalis*, leukocyte esterase, nitrite, protein, occult blood, RBC count, WBC count, and epithelial cell count. Leukocyte esterase, protein, and occult blood were coded as ordinal integers from 0 to 4 (negative, trace, 1+, 2+, and 3+), 5 (negative, trace, 1+, 2+, 3+, and 4+), and 5 (negative, trace, 1+, 2+, 3+, and 4+), respectively. Positive and negative results for the presence of nitrites and *T*. *vaginalis* were coded as 1 and 0, respectively. RBC, WBC, and epithelial cell counts were recorded on the basis of the number of cells in a specimen. Sex and age were recorded during the urinalysis test. The Chang Gung Medical Foundation Institutional Review Board approved this study (IRB no. 201601403B0), granting a waiver of patient consent.

### Strategy and model development

**Figure 6:**
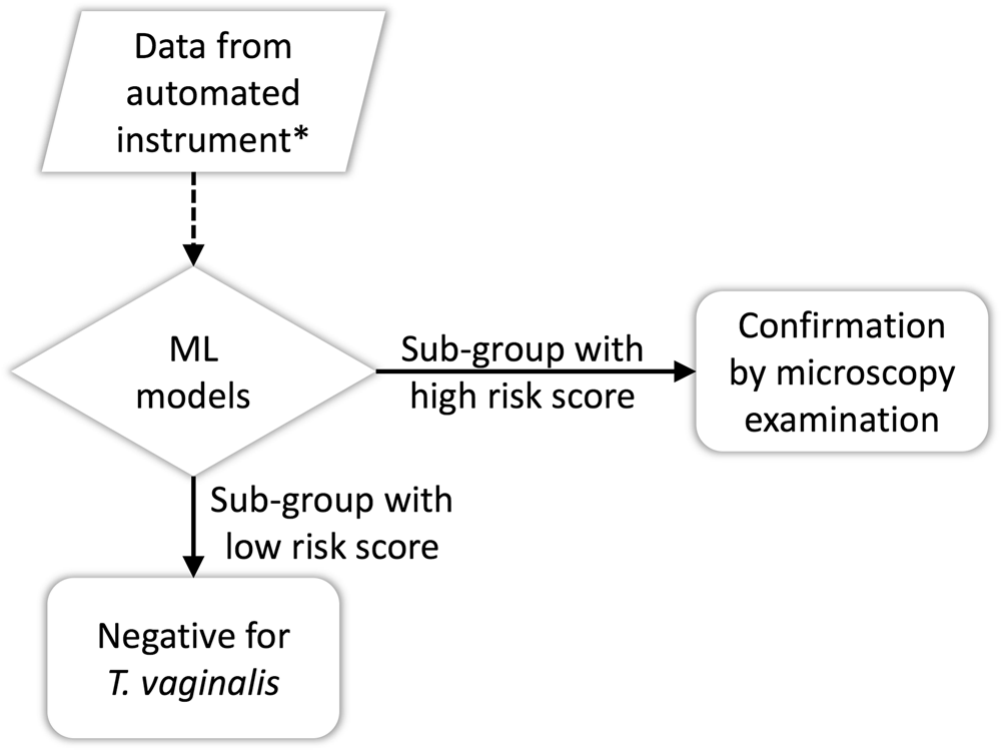
Flowchart of the machine learning-based strategy for *Trichomonas vaginalis* detection in urine. ML: Machine learning. *The data included leukocyte esterase, nitrite, protein, occult blood, red blood cell count, white blood cell count, and epithelial cell count.

Figure 6 presents a flowchart of the proposed ML-based strategy. Urinalysis data obtained from automated instruments were first analyzed using ML models. The ML models scored the *T*. *vaginalis* infection risk of each specimen and selected the risky subgroup for confirmation through microscopic examinations. Supplementary Figure 3 shows the framework of ML model development and validation. Patients satisfying the inclusion criteria were randomly assigned to one of five folds. We used a 5-fold cross-validation approach to train (four folds) and test (one fold) the models. To analyze imbalanced data, we randomly selected 10 sets of controls in each round of cross validation, matching the number of cases and age, and generated 10 training datasets by using one set of controls and all cases. Another 5-fold cross-validation process was conducted to tune the classification model in the training step. To account for sex-specific differences, we trained separate models for men and women.

We used logistic regression, SVM with a radial basis function kernel, and random forest to construct predictive models for *T*. *vaginalis*. Logistic regression measures the relationship between categorical dependent variables and one or more independent variables by using probability scores as the predicted values of the dependent variables^51^. SVM is a data-mining method that constructs a classification model for a binary-class problem. It uses nonlinear mapping to transform the data into a higher dimension. Through an appropriate nonlinear mapping to a sufficiently high dimension, data from two classes are separated by a hyperplane^52^. Random forest is an ensemble classifier proposed by Breiman^43^, comprising many classification trees, the bagging idea, and random selection of features. The frequency of a feature’s appearance in classification trees represents the importance of the feature. The models were constructed and tested using R software (version 3.3.2, R Foundation for Statistical Computing, http://www.r-project.org/) with the caret package^53^.

### Evaluation methods

We used the testing set, consisting of one fold of the individuals, to validate the classification models trained by three classifiers with the other four folds of the individuals (Supplementary Fig. 3). The outputs of the model were considered as the risk scores of *T*. *vaginalis* infection. The AUC and Lift values were used for evaluating model performance. The AUC is a performance measurement for classification problem at various thresholds settings^54^, representing how much the model is capable of distinguishing between classes, interpreted in the ranges of 0.9–1, 0.8–0.9, 0.7–0.8, 0.6–0.7, and 0.5–0.6 as representing excellent, very good, good, sufficient, and fail model, respectively^37,38^. The Lift is a measure of the effectiveness of a predictive model calculated as the ratio between the results obtained with and without the predictive model^54^. For example, suppose a population has an average disease prevalence rate of 5% but a prediction model has identified a high-risk group with a disease rate of 40%. Then, that high-risk group would have a Lift of 8.0 (40% divided by 5%).

### Variable importance

We evaluated the importance of variables by the mean decrease in Gini^43^. The Gini impurity is a measure of how often a randomly chosen element from the set would be incorrectly labeled if it was randomly labeled according to the distribution of labels in the subset, computed by summing the probability of an item with a label being chosen times the probability of a mistake in categorizing that item^55^. A split of a node in a tree is made when the Gini impurity criterion for the two descendant nodes is less than that for the parent node. Subsequently, the Gini decreases for each variable over all trees in the forest are summed to determine the variable importance. A higher mean decrease in Gini value represents the greater importance of the variable.

### Cost-effectiveness analysis

To systematically determine the trade-off between the number of specimens tested and the sensitivity of the classification models, we calculated the sensitivity level at different percentages of specimen tests. Furthermore, we defined the ICER as the number of specimens microscopically examined divided by the number of positive cases found, demonstrating the cost (number of specimens tested) at different levels of effectiveness (number of positive cases found).

### Statistical analysis

The Student t test, Mann–Whitney U test, and Pearson chi-squared test were used for continuous, ordinal, and categorical data, respectively. Analysis of variance was performed to determine the performance differences among classifiers. All analyses were performed using R software. All statistical tests were two-sided with an α error level of 0.05.

## Conclusion

The ML-based *T*. *vaginalis* detection strategy provides a cost-effective means of selecting urine specimens for microscopic examinations, by using multiple urinalysis data obtained from automated instruments. The model can be used in other laboratories that encounter the same problems because of the introduction of automated instruments for urine sediment examinations.

## Supplementary information

Supplementary Tables and Figures

## Data Availability

Data are available from the Ethics Committee of the Chang Gung Memorial Hospital for researchers who meet the criteria for access to confidential data. Requests for the data may be sent to the Chang Gung Medical Foundation Institutional Review Board, Taoyuan City, Taiwan (e-mail: irb1@cgmh.org.tw).
