# Supplementary material for: Increase Trichomonas vaginalis detection based on urine routine analysis through a machine learning approach
Source: Sci Rep. 2019 Aug 19;9:11074. doi: 10.1038/s41598-019-47361-8 (PMC6698480; doi:10.1038/s41598-019-47361-8)
Supplement: Supplementary file 1 — Supplementary Tables and Figures [file 41598_2019_47361_MOESM1_ESM.pdf]

Increase *Trichomonas vaginalis* detection based on urine routine analysis through a machine learning approach

Hsin-Yao Wang[1,2,3]†, Chung-Chih Hung[1,4,5,6]†, Chun-Hsien Chen[1,7], Tzong-Yi Lee[8,9,10,11], Kai-Yao Huang[10], Hsiao-Chen Ning[1,4], Nan-Chang Lai[1], Ming-Hsiu Tsai[5], Li-Chuan Lu[12], Yi-Ju Tseng[1,7,13]*, Jang-Jih Lu[1,3,4]*

[1] Department of Laboratory Medicine, Chang Gung Memorial Hospital at Linkou, Taoyuan, Taiwan

[2] Ph.D. Program in Biomedical Engineering, Chang Gung University, Taoyuan, Taiwan

[3] School of Medicine, Chang Gung University, Taoyuan, Taiwan

[4] Department of Medical Biotechnology and Laboratory Science, Chang Gung University, Taoyuan, Taiwan

[5] Graduate Institute of Technological and Vocational Education, National Taipei University of Technology, Taipei, Taiwan

[6] Department of Laboratory Medicine, Taipei Hospital, Ministry of Health and Welfare, New Taipei City, Taiwan

[7] Department of Information Management, Chang Gung University, Taoyuan, Taiwan

[8] Department of Computer Science & Engineering, Yuan Ze University, Taoyuan, Taiwan

[9] Innovation Center for Big Data and Digital Convergence, Yuan Ze University, Taoyuan, Taiwan

[10] Warshel Institute for Computational Biology, Chinese University of Hong Kong, Shenzhen, China

[11] School of Science and Engineering, Chinese University of Hong Kong, Shenzhen, China

[12] Department of Pathology, National Defense Medical Center, Division of Clinical Pathology, Tri-Service General Hospital, Taipei, Taiwan

[13] Research Center for Emerging Viral Infections, Chang Gung University, Taoyuan, Taiwan

† Hsin-Yao Wang and Chung-Chih Hung contributed equally to this manuscript.

*To whom correspondence should be addressed: YJ Tseng and JJ Lu

Supplementary Table 1. Performance of the Single-Variable Model for *Trichomonas vaginalis* Detection.

	AUC (mean±SD[1])	
**Urinalysis variable**	**Men**	**Women**
**Leukocyte esterase**	0.721±0.037	0.819±0.017
**Protein**	0.603±0.053	0.563±0.027
**Occult blood**	0.545±0.073	0.599±0.023
**Red blood cell**	0.606±0.076	0.655±0.017
**White blood cell**	0.794±0.049	0.800±0.022
**Epithelial cell**	0.781±0.039	0.813±0.014

[1]SD: Standard deviation.

Supplementary Table 2. Detailed Performances and Incremental Cost-Effectiveness Ratios of Various Strategies.

		**Specimens**	**5%**	**10%**	**25%**	**50%**	**75%**
**Women**	**Random Forest**	Sensitivity%	42.5	59.6	83.5	96.2	99.1
		Specificity%	95.1	90.1	75	50	24.6
		ICER	99.4	140.3	249.1	431.6	630.5
	**SVM**	Sensitivity%	39.4	57.1	80.4	95.2	98.3
		Specificity%	95.1	90.1	75.1	50.1	25
		ICER	107.5	146.6	258.5	435.2	632.3
	**Regression**	Sensitivity%	42	55	80.2	96.6	99.4
		Specificity%	95.1	90.1	75.1	50.1	25
		ICER	99.3	151.7	258.9	429	625.2
**Men**	**Random Forest**	Sensitivity%	42.3	55.5	74.2	89.3	98.2
		Specificity%	95	90	74.9	49.9	24.3
		ICER	566.9	846.5	1533.3	2553.7	3481.8
	**SVM**	Sensitivity%	38.5	52.8	67.3	84.5	95
		Specificity%	95	90	75	50	25
		ICER	616.9	876.9	1714.8	2710.8	3577
	**Regression**	Sensitivity%	35.1	53.6	73.6	81.8	89.2
		Specificity%	95	90	75	50	25
		ICER	803.5	898.5	1566.8	2794.1	3820.8

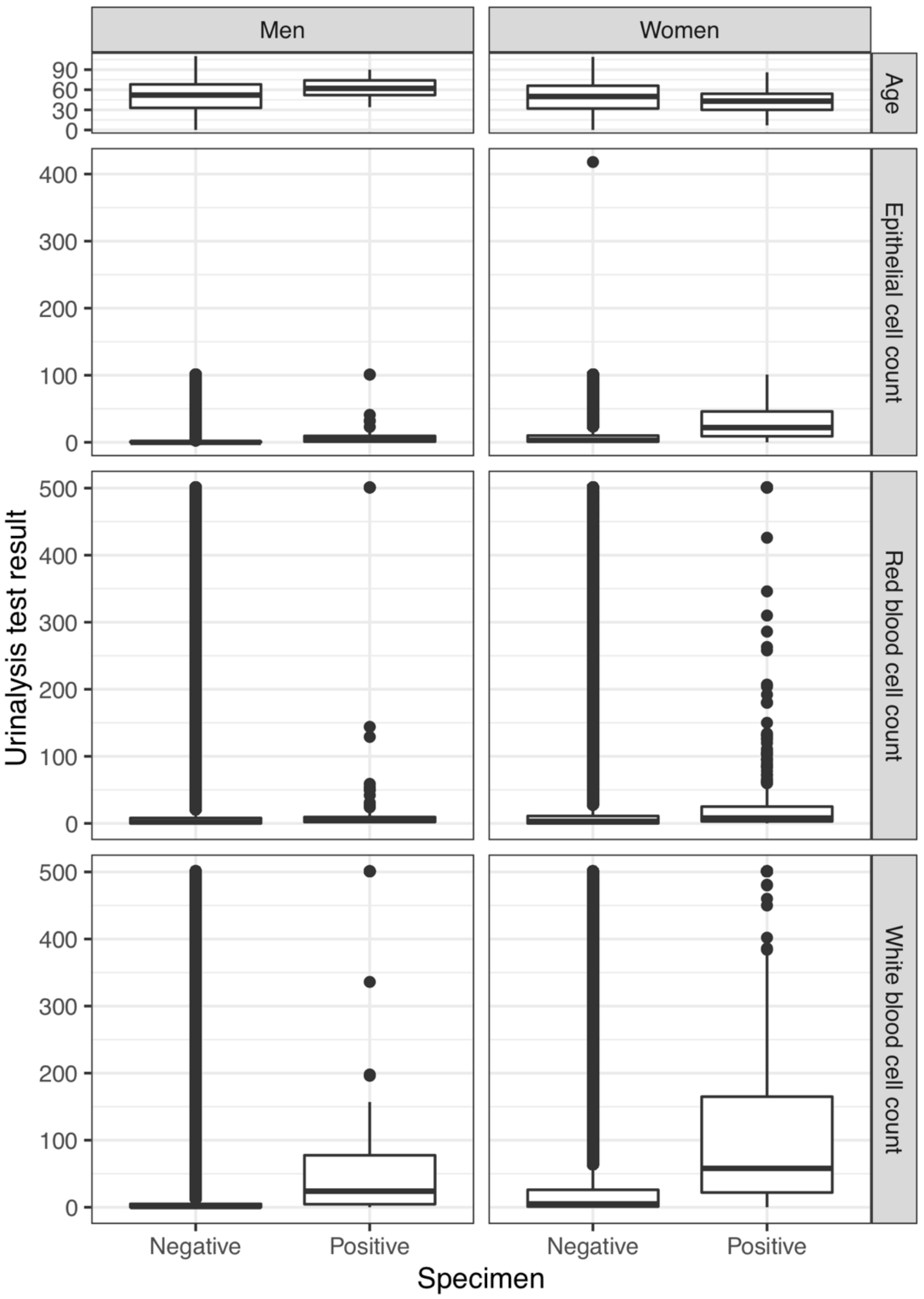

Supplementary Figure 1. The distributions of continuous features of urinalysis test.

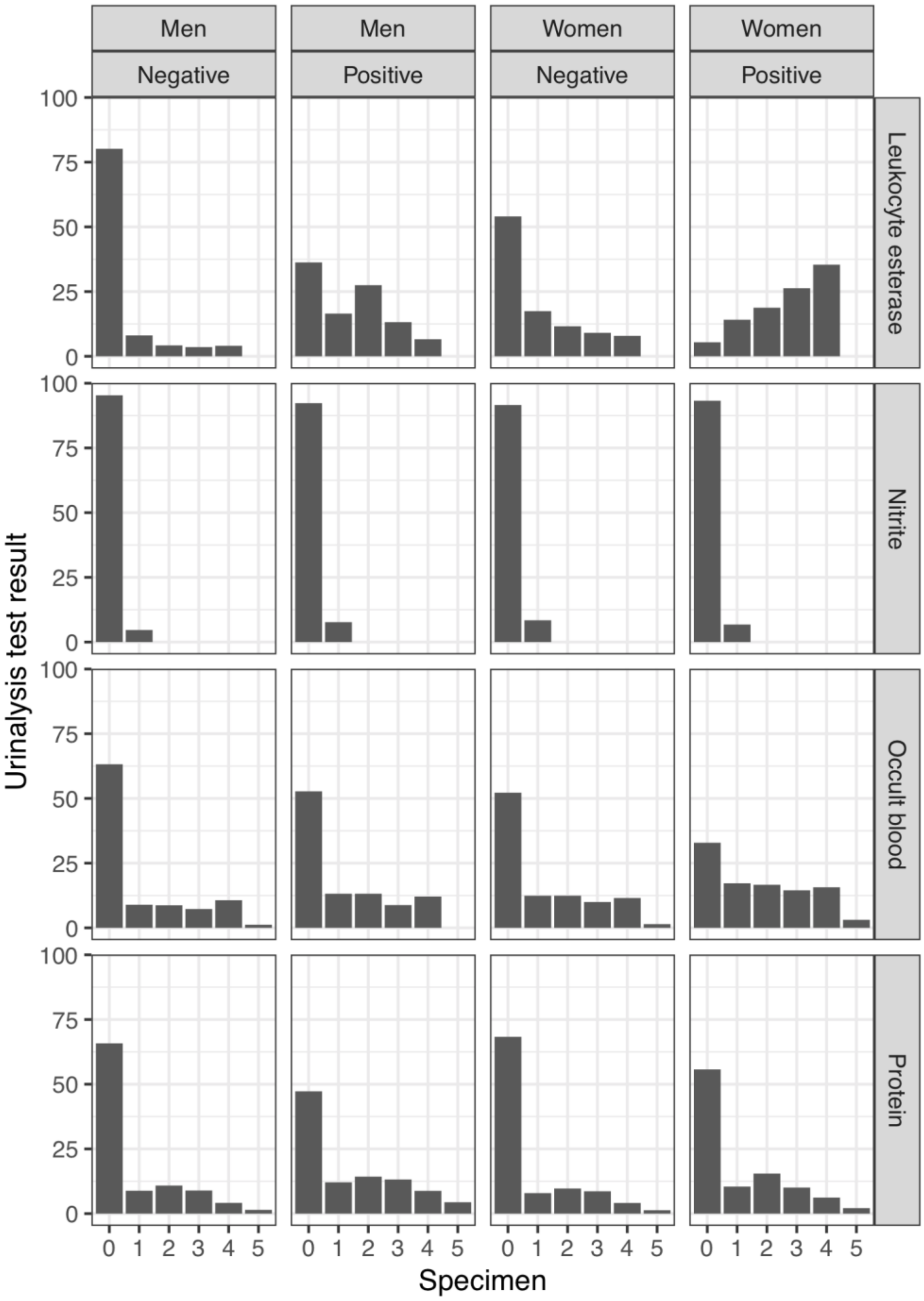

Supplementary Figure 2. The distributions of noncontinuous features of urinalysis test.

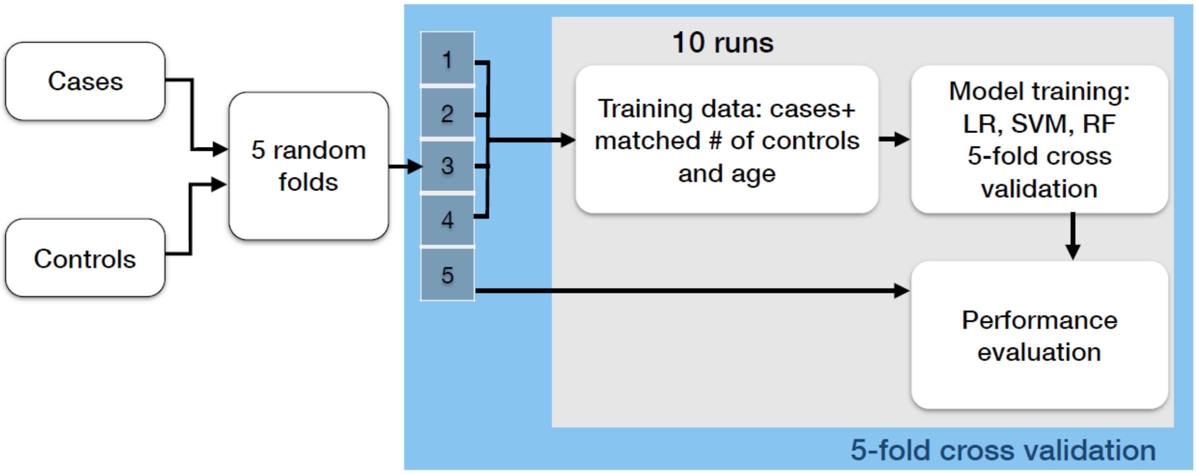

Supplementary Figure 3. Framework for model development and validation. LR: Logistic regression; SVM: Support vector machine; RF: Random forest.
